# H_2_O_2_-induced oxidative stress improves meat tenderness by accelerating glycolysis via hypoxia-inducible factor-1α signaling pathway in postmortem bovine muscle

**DOI:** 10.1016/j.fochx.2022.100466

**Published:** 2022-10-04

**Authors:** Cheng Chen, Zhaobin Guo, Xixiong Shi, Yuxuan Guo, Guoyuan Ma, Jibing Ma, Qunli Yu

**Affiliations:** College of Food Science and Engineering, Gansu Agricultural University, Lanzhou 730070, China

**Keywords:** Glycolysis, Hypoxia-inducible factor-1α, Reactive oxygen species, Signaling pathway, Tenderness, Bovine muscle

## Abstract

Reactive oxygen species (ROS) affect meat quality through multiple biochemical pathways. To investigate the effect of ROS on postmortem glycolysis and tenderness of bovine muscle, ROS content, glycolytic potential, glycolysis rate-limiting enzyme activities, expression of hypoxia-inducible factor-1α (HIF-1α), phosphatidylinositol 3-kinase (PI3K), serine-threonine kinase (AKT), phosphorylated AKT (p-AKT), and tenderness were determined in the H_2_O_2_ group and control group. Results showed that the H_2_O_2_ group exhibited significantly higher ROS content within 48 h, coupled with increased glycolytic potential, pH decline, hexokinase (HK), and phosphofructokinase activities (PFK) early postmortem. These were attributed to ROS-induced PI3K/AKT signaling pathway activation and resultant HIF-1α accumulation. Moreover, shear force in the H_2_O_2_ group reached the peak 12 h earlier and decreased obviously after 24 h, accompanied by a significantly higher myofibril fragmentation index (MFI). These findings suggested that ROS drive HIF-1α accumulation by activating PI3K/AKT signaling pathway, thereby accelerating glycolysis and tenderization of postmortem bovine muscle.

## Highlights

• Reactive oxygen species enhance the glycolytic potential of postmortem muscle.
• Reactive oxygen species activate hexokinase and phosphofructokinase early postmortem.
• Reactive oxygen species drive the hypoxia-inducible factor-1α accumulation.
• Hypoxia-inducible factor-1α contributes to enhancing glycolytic potential.
• Elevated glycolytic potential accelerates tenderization of muscle.

## 1 Introduction

After slaughtering, muscle cells are subjected to hypoxia stress, which shifts their energy metabolism toward the glycolytic pathway (Honikel, 2014). There is a range of biochemical changes occurring in the process of postmortem glycolysis. Among them, pH decline and glycogen consumption are closely associated with such attributes of meat quality as texture, color, flavor, and water-holding capacity (Kamenik et al., 2021, Li et al., 2020). It is well known currently that even a slight change in glycolytic flux can lead to a significant variation in meat properties (Chen et al., 2019, Honikel, 2014). For example, inadequate glycolysis leads to dark, firm, and dry (DFD) meat, which is characterized by high pH, unacceptable dark color, firm texture, and stale off-flavor (Chen et al., 2019, Zhang et al., 2019). A previous study found that a high amylose content in the diet decreases the postmortem glycolysis rate and further reduces drip loss and cooking loss of pork (Li et al., 2017). While preslaughter stress tends to cause excessive glycolysis of muscle that accelerates pH decline and protein denaturation during postmortem aging (Li et al., 2016, Sun et al., 2019). However, the underlying regulatory mechanisms of postmortem glycolysis remain incompletely understood.

In recent years, reactive oxygen species (ROS) have attracted much interest from researchers due to their role in regulating the transformation of muscle to meat. Constantly generated from the mitochondrial respiratory chain under hypoxic conditions, ROS may affect meat quality by mediating various biochemical pathways (Angelova and Abramov, 2016, Lana and Zolla, 2015, Turpaev, 2002). Recently, Yan, Chen, Huang, Huan, and Li (2022) suggested that excess ROS can impair meat quality by aggravating lipid peroxidation and drop loss. Conversely, Chen et al. (2021) demonstrated that ROS accumulation ameliorates the meat texture via inducing satellite cell proliferation and collagen synthesis in grass carp muscle. In addition, several studies suggested that ROS, especially hydrogen peroxide (H_2_O_2_), acts as a secondary messenger molecule to activate apoptosis and caspase-3, which is conducive to the tenderization of lamb and bovine muscle (Herrera et al., 2021, Ma et al., 2022, Wang et al., 2018). According to the above literature, there is inconsistency in describing the influence of ROS on meat quality. Interestingly, however, it was generally found in the above studies that ROS contributed to pH decline early postmortem. A recent study suggested that ROS can promote pH decline by accelerating postmortem glycolysis in broiler meat (Xing, Chen, Li, Zhang, & Gao, 2021). Notably, rapid glycolysis can also result in a lower shear force in the process of postmortem aging, thereby improving meat tenderness (Kamenik et al., 2021). Nevertheless, the role and underlying mechanism of ROS in postmortem glycolysis remain unclear. It is widely known in the medical field that the mitochondrial respiratory chain releases H_2_O_2_ to stimulate and stabilize the expression of hypoxia-inducible factor-1α (HIF-1α), which is crucial for the response to hypoxic stress through triggering glycolysis (Paik et al., 2017, Pinheiro et al., 2010, Turpaev, 2002). Another study demonstrated that HIF-1α is effective in increasing glycolytic flux in postmortem porcine muscle (Li et al., 2017). Despite this, there is still no firm evidence suggesting whether ROS can regulate glycolysis and further affect tenderization through the HIF-1α pathway in postmortem muscle.

HIF-1α is known as a major transcriptional regulator in mammal skeletal muscle that can regulate several fundamental pathways in the responses of cells to hypoxic and oxidative stress (Paik et al., 2017). As suggested by the evidences obtained from prior studies, HIF-1α can bind to hypoxia response element (HRE) in the nucleus, thus up-regulating gene expression and enzymic activation for nearly 90 % of the glycolytic enzymes, particularly for such rate-limiting enzymes as hexokinase (HK), phosphofructokinase (PFK) and pyruvate kinase (PK) (Basse et al., 2017, Tang and Zhao, 2020). Under aerobic conditions, HIF-1α tends to be targeted by prolyl hydroxylases for ubiquitination and rapid proteasomal degradation (Pinheiro et al., 2010). While under hypoxic conditions, ROS-induced oxidative stress plays an indirect role in stabilizing HIF-1α through the stimulation of the phosphatidylinositol 3-kinase (PI3K)/serine-threonine kinase (AKT) signal path (Moldogazieva et al., 2020, Paik et al., 2017). It has been reported that H_2_O_2_ induces the expression of PI3K that targets AKT for phosphorylation (Angeloni et al., 2011). More importantly, PI3K and phosphorylated AKT (p-AKT) are essential for the binding of HIF-1α to HRE and the inhibition of HIF-1α degradation, respectively, which is necessary for activating those critical glycolytic enzymes (Li et al., 2017). Based on these studies, it is hypothesized in this study that ROS functions as upstream messenger molecules to stabilize HIF-1α through the PI3K/AKT signaling pathway, thus activating glycolytic enzymes and up-regulating glycolysis during the postmortem aging of bovine muscle. Therefore, this study is aimed to establish whether ROS exerts an effect on the modulation of glycolysis and subsequent tenderization in postmortem bovine muscle through the PI3K/AKT-HIF-1α pathway, which might facilitate the understanding of the latent mechanisms of meat quality development.

## 2 Materials and methods

### 2.1 Sample collection and treatment

A total of six 3-year-old bulls (Chinese yellow cattle × Angus, average bodyweight 450 ± 20 kg) from the same livestock system were slaughtered at a local commercial meat processing company (Qilian Pastoral Food Industry Co. ltd., Zhangye City, Gansu province, China), in line with the Chinese National Standards for Operating Procedures of Cattle Slaughter. Immediately after skinning, each *longissimus lumborum* (LL) muscle was removed from the left side of the carcass and cut into five pieces sized 10 × 10 × 2 cm (length × width × thickness). All the muscle pieces were mixed to obtain a total of thirty same-sized samples, which were then randomly divided into two equal groups (fifteen replicates per group) and treated. More specifically, one group was injected with 200 μM H_2_O_2_ at a ratio of 10:1 (w/v; meat: solution) and treated as the H_2_O_2_ group, while the other group was injected with 0.9 % saline at the same ratio and treated as the control group. H_2_O_2_ is commonly used to induce oxidative stress in cells (Pinheiro et al., 2010). All the muscle pieces were individually vacuum-packed and stored at 4 °C for 0.5, 2, 6, 12, 24, 36, and 48 h, respectively. After the pH value and tenderness parameters were determined at each end of the time point, appropriate samples were collected from each muscle piece and instantly frozen in liquid nitrogen for biochemical analysis.

### 2.2 ROS detection

ROS content was analyzed using a fluorescence assay (Zhang et al., 2019). In brief, 3 g of frozen LL sample was homogenized twice in 15 mL of 50 mM pre-cooling potassium phosphate buffering solution (pH 7.4) at 12,000 rpm for 20 s at a 10 s interval and subjected to centrifugation at 5,000 × *g* for 20 min at 4 °C. Then, the supernate was cultivated under 37 °C for 20 min in an equivalent volume of solution [10 μM 2′,7′-dichlorohydrofluorescein diacetate (DCFH-DA), 10 mM Tris-HCl, 15 mM sucrose, 0.15 mM EDTA-2Na, 0.9 % (w/v) NaCl, pH 7.4]. The level of fluorescence intensity before and after incubation was measured at 450 nm using a fluorescence spectrophotometer (F-4700, Hitachi, Tokyo, Japan). ROS content was expressed as the ratio of the difference in fluorescence intensity before and after incubation to protein concentration to incubating time.

### 2.3 Glycogen, lactate, and glycolytic potential analysis

Glycogen content was identified using the approach proposed by Chen et al. (2019). In brief, 0.5 g of minced LL sample was mixed with 1.5 mL of 2 M NaOH and then kept boiling for 20 min. Subsequently, the cooled mixture was mixed with 8 mL of ultrapure water and subjected to centrifugation at 5,000 × *g* for 10 min. Finally, the supernate was utilized to identify glycogen level with the assistance of a commercial glycogen assay kit (Sigma‐Aldrich, St. Louis, MO, America).

Lactate content was measured following the method described by Li et al. (2016). In general, 0.5 g of minced LL sample was homogenized in 4.5 mL of ice-cold saline at 12,000 rpm for 30 s. After the centrifugal process at 3,000 × *g* for 15 min under 4 °C, the supernate was collected and applied to identify lactate level with the assistance of a commercial lactate assay kit (Sigma‐Aldrich).

Glycolytic potential was evaluated based on the formula suggested by Li et al. (2017), where Glycolytic potential = 2 × glycogen + lactate.

### 2.4 pH determination

The pH of the LL sample was determined using a portable pH meter (HI 98163, Hanna Instruments, Italy). The needle electrode was calibrated at room temperature (25 °C) before each determination. Determination was conducted by inserting the pH probe into samples at random points with a similar depth.

### 2.5 Enzyme activities determination

The extracts intended for the detection of HK, PFK, and PK activities were prepared using the method proposed by Wang et al. (2020). To be specific, 1 g of frozen LL sample was added into 9 mL of 10 mM cold homogenizing solution (80 mM K_2_HPO_4_, 15 mM KH_2_PO_4_, 25 mM KCl, 1.2 M NaCl, pH 7.4) and then homogenized at 12,000 rpm for 30 s in an ice bath. Subsequently, the homogenate was subjected to centrifugation at 13,000 × *g* for 15 min under 4 °C. Afterward, the supernate was collected to detect the activities of HK, PFK, and PK using commercial ELISA kits (Jiancheng Bioengineering Institute, Nanjing, China). Moreover, protein concentration was measured using a BCA protein assay kit (Thermo Scientific, Rockford, IL, America), while enzyme activities were normalized respectively according to the total protein concentration.

### 2.6 Western blotting analysis

The proteins of the LL sample were extracted using the method put forward by Li et al. (2017)**.** After the separation of proteins by SDS-PAGE (10 % separation gel and 5 % concentration gel), the target proteins (50 μg) were transferred onto polyvinylidene difluoride (PVDF) films (Millipore, Bedford, MA, America). Then, PVDF films were subjected to blockade in 5 % fat-free milk in Tween 20 (TBS-T) solution (0.1 % Tween-20, 150 mM NaCl, 10 mM Tris, 5 mM KCl, pH 7.4) under ambient temperature and then incubated for 12 h under 4 °C using a variety of different primary antibodies: anti-HIF-1α antibody (1:500), anti-PI3K antibody (1:500), anti-AKT antibody (1:500), anti-p-AKT (Ser473) antibody (1:500), and anti-Actin antibody (1:3,000) (Cell Signaling Technology, Beverly, MA, America). Posterior to cleaning three times (10 min per time) with TBS-T solution, the films were cultivated for 1 h under ambient temperature using anti-rabbit horseradish peroxidase-conjugated second antibody (1:5,000) (Abcam, Cambridge, UK). Then, the films were cleaned thrice in TBS-T solution again and the proteins were visualized using the ECL assay kit on a ChemiDoc MP imaging system (Bio-Rad, Hercules, America). The expressing levels of proteins were subjected to quantification via the Quantity-One program 4.6.2 (Bio-Rad, America) and afterward normalized to actin.

### 2.7 Shear force and myofibril fragmentation index (MFI) determination

Shear force was measured as previously described by Wang et al. (2018). Briefly, the LL sample was packed in polyethylene bags in an 80 °C water bath and cooked to an internal temperature of 75 °C. After cooling to room temperature, three to six cylindrical samples (diameter 1.27 cm, height 2 cm) were obtained parallel to muscle fibers using a borer. Subsequently, each sample was sheared perpendicular to the muscle fibers with a Warner-Bratzler tenderometer (C-ML3B, Harbin, China). The peak force was regarded as the shear force and the result was expressed by Newton (N).

MFI was assayed according to the procedure described by Chen et al. (2019). 5 g of LL sample was homogenized at 10,000 rpm for 60 s with an addition of 45 mL of cold buffer solution (100 mM KCl, 20 mM K_3_PO_4_, 1 mM EDTA, 1 mM MgCl_2_, pH 7.0), and then centrifuged twice at 1,000 × *g* for 20 min. The precipitate was resuspended with the buffer solution at a final protein concentration of 0.5 mg/mL. Absorbance was detected at a wavelength of 540 nm using a spectrophotometer (UV-2401, Shimadzu, Japan). MFI was expressed as absorbance multiplied by 200.

### 2.8 Statistics

Data were studied by one-way ANOVA via the general linear model (GLM) of the SPSS 20.0 program (SPSS Inc., America). Duncan's multiple range test (*P* < 0.01) was performed to compare the significance of the difference among mean values. The carcass was a random effect, and the treatments and storage time were fixed effects. Results were expressed as mean ± standard error from six biological replicates.

## 3 Results and discussion

### 3.1 H_2_O_2_-induced oxidative stress accelerated ROS generation

**Fig. 1:**
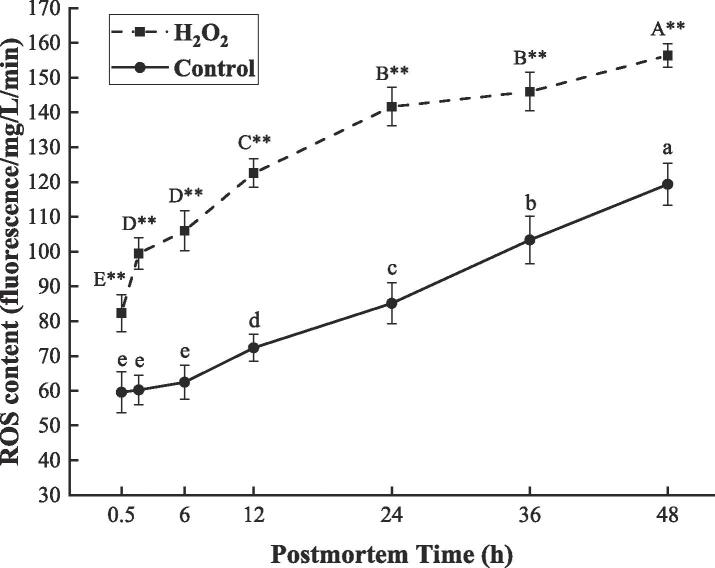
Changes in ROS content of bovine muscle when stored at 4 °C for 0.5, 2, 6, 12, 24, 36, and 48 h after treatment with 200 μM H_2_O_2_ (H_2_O_2_ group) and saline (control group). Data were expressed as mean ± standard error (n = 6). The capital letters and lowercase letters represent the significant difference (*P* < 0.01) in the same group at different times, respectively. ** (*P* < 0.01) represents the difference between the two groups at the same time point. ROS: reactive oxygen species.

After slaughter, muscle cells inevitably produce ROS under conditions of hypoxic stress, which induce oxidative stress and cause further accumulation of ROS (Angelova and Abramov, 2016, Chen et al., 2020). To confirm whether oxidative stress could promote mitochondrial ROS accumulation, the muscle samples were treated with 200 μM H_2_O_2_, with the ROS content detected during the postmortem storage. As shown in Fig. 1, ROS content was significantly increased by 1.9 times in the H_2_O_2_ group at 0.5–48 h (*P* < 0.01). In contrast, the control group showed an increase in ROS content after 6 h (*P* < 0.01). Notably, ROS content was found to be 1.4 times greater in the H_2_O_2_ group in contrast to the control at 0.5 h and it remained significantly greater in the H_2_O_2_ group in contrast to the control throughout the study period (*P* < 0.01). Consistent with our results, Yan et al. (2022) found that ROS content of broiler thigh muscle injected with 2.5 % H_2_O_2_ pre-slaughter is approximately 2 times higher than that of the control. The above results demonstrated that H_2_O_2_-induced oxidative stress can enhance the generation of ROS in the postmortem bovine muscle during the early postmortem period.

Under hypoxic conditions, the mitochondrial respiratory chain acts as a hypoxia sensor through a constant release of ROS. In this case, a high level of ROS leads to an increase in oxidative stress, thus triggering the further production of ROS through multiple pathways (Angelova and Abramov, 2016, Basse et al., 2017). At present, it has been reported that H_2_O_2_-induced oxidative stress can promote the release of lysosomal ferric iron into the cytoplasm and catalyze intracellular ROS production through the Fenton-like reaction (Zhang, Yu, Han, Han, & Han, 2020). Meanwhile, the muscle antioxidant system is disrupted as the additional production of ROS contributes to increased oxidative stress (Wang, et al., 2018). These findings may help explain the result obtained in this study that exogenous H_2_O_2_ treatment increased the initial level of ROS and resulted in a vicious circle of ROS over the study period (Fig. 1). Besides, it has been demonstrated that endogenous antioxidants (such as α-tocopherol, superoxide dismutase, catalase, etc.) lead to a significant reduction in the ROS content at the early postmortem stage, while the ROS scavenging ability decreases with the extension of postmortem aging time (Wang et al., 2018, Yan et al., 2022). These findings suggested that ROS accumulation is an inevitable event in the postmortem muscle.

### 3.2 ROS enhanced the glycolytic potential and accelerated the pH decline early postmortem

**Fig. 2:**
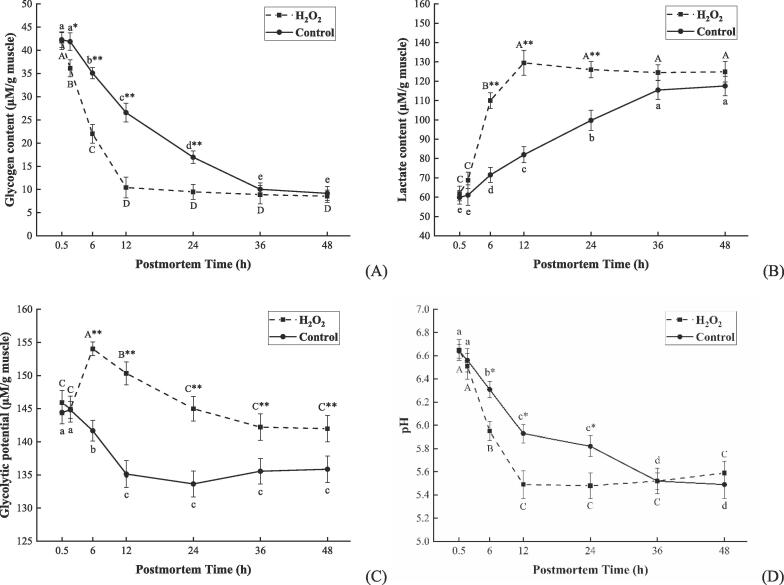
Changes in glycogen content (A), lactate content (B), glycolytic potential (C), and pH (D) of bovine muscle when stored at 4 °C for 0.5, 2, 6, 12, 24, 36, and 48 h after treatment with 200 μM H_2_O_2_ (H_2_O_2_ group) and saline (control group). Data were expressed as mean ± standard error (n = 6). The capital letters and lowercase letters represent the significant difference (*P* < 0.01) in the same group at different times, respectively. * (*P* < 0.05) and ** (*P* < 0.01) represent the difference between the two groups at the same time point, respectively.

The oxygen supply to muscle cells is disrupted after slaughtering, thus resulting in hypoxic stress. ROS play an important role in cell response to hypoxia, which activate multiple signaling pathways that maintain cellular energy supply through a shift of oxidative respiration to aerobic glycolysis (Angelova and Abramov, 2016, Paik et al., 2017). However, it remains unclear how ROS relate to glycolysis in postmortem bovine muscle. Given the increased level of ROS in postmortem bovine muscle, it is hypothesized in this study that ROS are involved in the regulation of muscle glycolysis. As shown in Fig. 2 A, glycogen was consumed and reduced to the same level within 48 h in both groups (*P* < 0.01). Notably, it took as little as 12 h for glycogen to decline to the lowest level in the H_2_O_2_ group, which is 24 h earlier than the control group. Meanwhile, glycogen content was observed to be remarkably lower in the H_2_O_2_ group in contrast to the control at 2–24 h (*P* < 0.01 or *P* < 0.05). Consistent with the consumption of glycogen, there was an increase in lactate accumulation for each group (Fig. 2 B). Despite the similar lactate levels shown by the two groups at 48 h, the H_2_O_2_ group reached the highest level at 12 h, which is 24 h earlier than the control group, indicating that ROS could improve the rate of glycolysis in the postmortem bovine muscle. The glycolytic potential is an indicator widely used to describe the glycolysis capacity of postmortem muscle, the increase in glycolytic potential implies an elevated glycolytic rate in postmortem muscle (Li et al., 2017). As shown in Fig. 2 C, the glycolytic potential of the H_2_O_2_ group increased substantially by 1.1 times at 2–6 h (*P* < 0.01) and maintained a significantly higher level in contrast to the control at 6–48 h (*P* < 0.01). Given that remarkable increased glycolytic potential at 2–6 h in the H_2_O_2_ group was accompanied by significantly elevated ROS content at 0.5–6 h (Fig. 1), it is suggested that elevated ROS levels enhanced glycolytic potential early postmortem. The changes in pH (Fig. 2 D) showed consistency with the lactate contents, while the H_2_O_2_ group showed a fast pace of pH decline, reaching a significant extent (*P* < 0.01) at 2–12 h and a lower level than in the control group at 6–24 h (*P* < 0.05).

Glycolysis has now been known as conducive to ATP production in the postmortem muscle in response to hypoxic stress, during which glycogen is decomposed into lactate (H^+^), thus reducing pH value (Zhang et al., 2019). It is also widely known that postmortem glycolysis can have a significant impact on meat quality by affecting the rate and extent of pH decline (Kamenik et al., 2021, Scheffler and Gerrard, 2007). Normally, the pH drops from 7.2 to around 5.4–5.7 and causes the gradual acidification of muscle during postmortem aging, which further affects the development of quality attributes including tenderness, color, and juiciness (Kamenik et al., 2021). For instance, when the pH falls below 6.0, proteolytic enzymes such as calpains and caspases are activated to degrade myofibrillar proteins, driving the onset of meat tenderization (Baron et al., 2021, Ma et al., 2022, Xing et al., 2021). Besides, the pH decline is also associated with protein denaturation, myoglobin oxidation, and water migration (Kamenik et al., 2021, Li et al., 2020). Therefore, a great effort has been made by researchers to identify the factors in regulating postmortem glycolysis, for example, diet types, preslaughter stress, and glycolytic enzyme activities (Li et al., 2017, Sun et al., 2019, Wang et al., 2020).

Recently, ROS are involved in the regulation of glycolysis when cells are subjected to hypoxic stress. As demonstrated by Pearlstein et al., 2002, Robinson et al., 2020, ROS accumulation is an earlier event upstream of glycolysis that can accelerate both glucose consumption and lactate accumulation in endothelial cells. Moreover, the ROS scavenger *N*-acetyl-l-cysteine (NAC) was shown to reduce the glycolytic rate in skeletal muscle by scavenging intracellular ROS (Chambers, Moylan, Smith, Goodyear, & Reid, 2009). This is coherent with our result that 200 μM of H_2_O_2_ treatment increased the level of ROS accumulation (Fig. 1), which enhanced the glycolytic potential while accelerating glycogen depletion, lactate accumulation, and pH decline (Fig. 2). Our results are also consistent with recent studies by Xing et al., 2019, Zhang et al., 2019, who found that oxidative stress accelerates pH decline by promoting glycolysis and the resultant lactate accumulation in postmortem muscle. As for the regulatory role of ROS in muscle glycolysis, Paik et al. (2017) indicated that ROS (especially H_2_O_2_) acts as a signaling molecule in the response to hypoxic stress by inducing the expression of the proteins upstream of glycolysis such as HIF-1α, HK, and glucose transporter-1. However, there remains little understood about the underlying mechanisms through which ROS regulates the postmortem glycolysis of bovine muscle.

### 3.3 The activity of rate-limiting enzymes of glycolysis was elevated by ROS

**Fig. 3:**
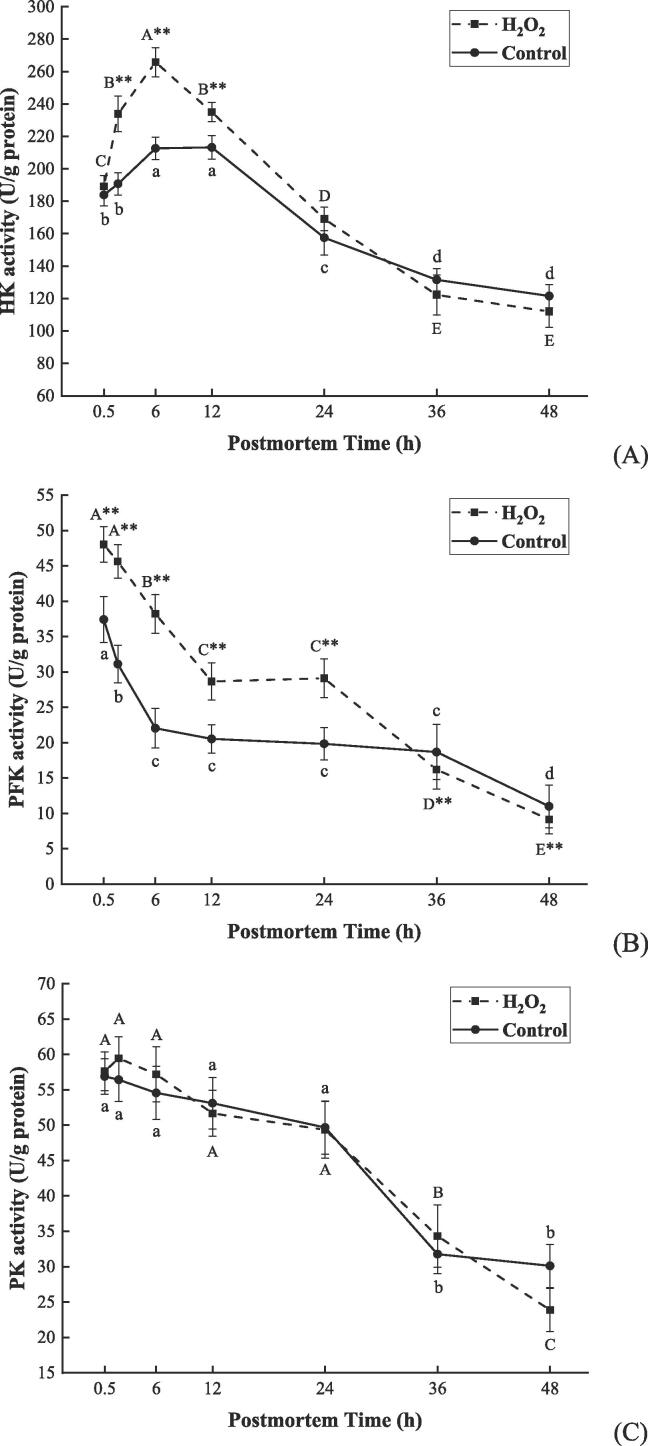
Changes in activities of HK (A), PFK (B), and PK (C) of bovine muscle when stored at 4 °C for 0.5, 2, 6, 12, 24, 36, and 48 h after treatment with 200 μM H_2_O_2_ (H_2_O_2_ group) and saline (control group). Data were expressed as mean ± standard error (n = 6). The capital letters and lowercase letters represent the significant difference (*P* < 0.01) in the same group at different times, respectively. ** (*P* < 0.01) represents the difference between the two groups at the same time point. HK: hexokinase, PFK: phosphofructokinase, and PK: pyruvate kinase.

The rate and extent of glycolysis are regulated by glycolytic enzymes, especially 3 rate-limiting enzymes HK, PFK, and PK (Basse et al., 2017). For this reason, the activities of HK, PFK, and PK were measured to further explore the effect of ROS on postmortem muscle glycolysis. As presented in Fig. 3 A, no remarkable diversity was observed in HK activity between the two groups at 0.5 h, despite it reaching the highest level at 6 h in the H_2_O_2_ group (265.67 U/g protein) and 12 h in the control group (213.23 U/g protein), respectively (*P* < 0.01). The H_2_O_2_ group exhibited a stronger HK activity than the control group at 2–12 h postmortem (*P* < 0.01), implying that ROS contributed to the increase of HK activity within 12 h postmortem. As shown in Fig. 3 B, PFK activity was significantly decreased during 48 h postmortem in both two groups (*P* < 0.01). However, the H_2_O_2_ group showed a stronger initial PFK activity (48.00 U/g protein) than the control group (37.40 U/g protein) at 0.5 h and maintained a significantly higher level within the next 24 h postmortem (*P* < 0.01). According to Fig. 3 C, PK activity displayed no remarkable changes between the two groups despite the decreasing trend exhibited at 24–48 h postmortem (*P* < 0.01). It is suggested that ROS is capable to enhance the activities of HK and PFK early postmortem.

HK, PFK, and PK are rate-limiting enzymes, the activities of which play a direct role in the rate of muscle glycolysis, thus further affecting the rate of pH decline (Basse et al., 2017, Chen et al., 2017). In this study, ROS was found effective in significantly enhancing the activities of HK and PFK respectively at 2–12 h and 0.5–24 h, which evidences their capability of improving glycolytic potential in the postmortem bovine muscle. It has also been revealed that ROS participates in the upregulation of the glycolytic rate in skeletal muscle cells by promoting the expression levels of HK and PFK (Paik et al., 2017, Pinheiro et al., 2010). ROS-induced HIF-1α stabilization can affect the glycolytic rate of cells by activating a range of glycolytic enzymes such as HK, PFK, PK, fructose-bisphosphate aldolase (ALD), enolase (ENO), and lactate dehydrogenase (LDH) (Moldogazieva et al., 2020). Another study also demonstrated that short-time exposure to ROS (H_2_O_2_) can promote HK and PFK activities while increasing glucose uptake and lactate production for muscle cells in vitro (Pinheiro et al., 2010). Although ROS has been widely known as cytotoxic effector molecules, Turpaev (2002) indicated the vital role played by ROS as signaling molecules in the activation of glycolysis by upregulating HIF-1α expression when cells sustain hypoxic stress. However, it remains unclear whether ROS could play a role in postmortem bovine muscle as mentioned above.

### 3.4 ROS-stimulated HIF-1α accumulation was sufficient to enhance glycolytic potential early postmortem

**Fig. 4:**
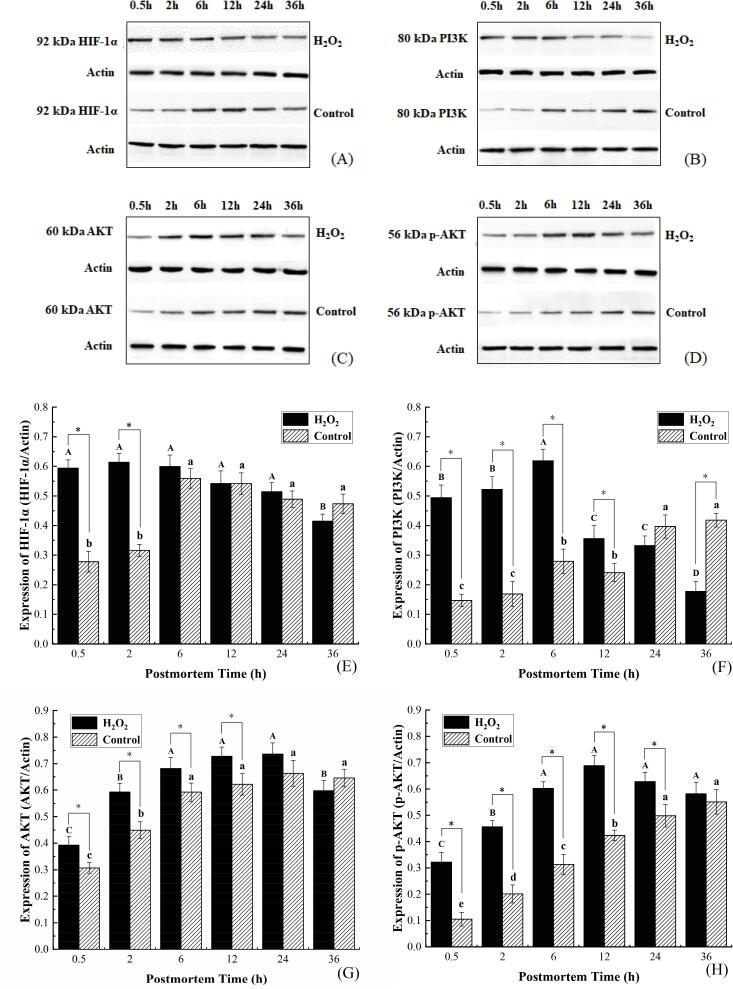
Representative western blots for HIF-1α (A), PI3K (B), AKT (C), and p-AKT (D) and quantitative analysis of HIF-1α (E), PI3K (F), AKT (G), p-AKT (H) protein levels of bovine muscle when stored at 4 °C for 0.5, 2, 6, 12, 24, and 36 h after treatment with 200 μM H_2_O_2_ (H_2_O_2_ group) and saline (control group). About 50 μg of protein was loaded per lane. Actin levels indicated equal protein loading per line. Protein expression levels are expressed as the proportion of abundance of the samples and actin. Data were expressed as mean ± standard error (n = 6). The capital letters and lowercase letters represent the significant difference (*P* < 0.01) in the same group at different times, respectively. * (*P* < 0.05) represents the difference between the two groups at the same time point. HIF-1α: hypoxia-inducible factor-1α, PI3K: phosphatidylinositol 3-kinase, AKT: serine-threonine kinase, and p-AKT: phosphorylated AKT.

Known as a key regulator of hypoxia-related energy metabolism, HIF-1α represents a transcription factor in regulating the activation of glycolytic enzymes (Paik et al., 2017). To better understand the correlation between ROS and HIF-1α in postmortem bovine muscle from a mechanism perspective, the expression levels of HIF-1α were identified and the outcomes are presented in Fig. 4 A and E. We can see from these figures that the expressing levels of HIF-1α were different between the two treatments, which was significantly up-regulated (*P* < 0.01) in the H_2_O_2_ group at the early postmortem stage (0.5–2 h), reaching a level that is 2.1 times and 1.9 times greater in contrast to the control at 0.5 h and 2 h, respectively (*P* < 0.05). Combined with the results shown in Fig. 2, Fig. 3, it can be demonstrated that ROS is essential for the accumulation of HIF-1α, which further regulates the activation of key glycolytic enzymes (HK and PFK) to enhance glycolytic potential early postmortem. Our results are partly consistent with those of Gao et al. (2019), who indicated that the greater glycolytic enzyme activity and glycolysis rate of yak meat within 24 h postmortem are attributed to its higher expression level of HIF-1α.

HIF-1α represents a major transcriptional regulator in the oxidative phosphorylation switch to glycolysis in mammal skeletal muscle (Basse et al., 2017, Turpaev, 2002). Under hypoxic conditions, HIF-1α is stabilized and transferred to the nucleus, thus activating the expression of various critical glycolysis-related proteins including rate-limiting enzymes (Nowicki & Gottlieb, 2015). It has now been demonstrated that the increase in the expression of LDH and glucose transporter-1 is induced by the upward trend of HIF-1α, which contributes to the generation of glycolytic products ATP and lactate (Sun, Guo, Lv, Shao, & Li, 2020). In addition, a recent study suggested that HIF-1α accumulation is dependent on the generation of ROS and acts as an upstream signaling factor in stabilizing HIF-1α expression (Basse et al., 2017). As revealed by Paik et al. (2017), NAC treatment can inhibit ROS generation significantly and disable ROS completely to induce HIF-1α accumulation. That is to say, ROS generation is a prerequisite for the stable expression of HIF-1α. Similarly, it has been discovered in the present research that, the higher expression level of HIF-1α at 0.5–2 h in the H_2_O_2_ group (Fig. 4 A and E) was coupled with the relatively higher level of ROS concentrations (Fig. 1), and further with the significantly higher activities of HK and PFK (Fig. 3 A and B) as well as glycolytic potential (Fig. 2 C). To sum up, the above-mentioned results demonstrated that ROS is required for HIF-1α accumulation, as it acts as a metabolic switch to enhance the glycolytic potential in the postmortem bovine muscle.

### 3.5 ROS up-regulated HIF-1α expression through PI3K/AKT signaling pathway

The PI3K/AKT signal path has been revealed to play an important role in HIF-1α expression under both hypoxic and oxidative stress conditions (Moldogazieva et al., 2020). However, there remains a lack of clarity on whether ROS could promote HIF-1α expression via the PI3K/AKT signal path in postmortem bovine muscle. As presented in Fig. 4 B and F, the expressing level of PI3K was remarkably up-regulated at 0.5–6 h in the H_2_O_2_ group, reaching a level that is 3.6 times, 3.1 times, 2.2 times, and 1.5 times greater in contrast to the control at 0.5 h, 2 h, 6 h, and 12 h, separately (*P* < 0.05). The expression of total AKT (Fig. 4 C and G) was significantly increased at 0.5–12 h in the H_2_O_2_ group, which is 1.3, 1.4, 1.2, and 1.2 times higher than the control at 0.5 h, 2 h, 6 h, and 12 h, respectively (*P* < 0.05). In the meantime, the expression of p-AKT (Fig. 4 D and H) was increased substantially at 0.5–12 h in the H_2_O_2_ group, which is 3.1 times, 2.3 times, 1.9 times, 1.6 times, and 1.3 times greater in contrast to the control at 0.5 h, 2 h, 6 h, 12 h, and 24 h, separately (*P* < 0.05). Similarly, a recent study found that stable accumulation of HIF-1α is accompanied by the up-regulated PI3K/AKT signal path in pork after 1 h postmortem (Liu et al., 2021). These results evidenced that ROS play a role in increasing the stimulation of the PI3K/AKT signal path in the postmortem bovine muscle. Furthermore, given the significantly higher expressing levels of HIF-1α in the H_2_O_2_ group (Fig. 4 A and E), it is suggested in this study that ROS can activate PI3K/AKT signaling pathway, thus enhancing HIF-1α expression in postmortem bovine muscle.

As one of the significant protein kinases, PI3K/AKT is widely recognized to play an essential role in the resistance of cells to oxidative stress, ischemic stress, and hypoxic stress (Tang & Zhao, 2020). It has been recently reported that a low concentration (100 μM) of H_2_O_2_ can induce the expression of PI3K that targets AKT for phosphorylation via phosphoinositide-dependent protein kinase-1 (PDK1) and that the phosphorylated AKT (p-AKT) is essential for HIF-1α accumulation (Angeloni et al., 2011, Moldogazieva et al., 2020). Despite the failure to reveal the underlying mechanism through which the P13K/AKT signaling pathway regulates the stable expression of HIF-1 α in this study, it has been indicated in a previous study that phosphorylated AKT can activate its downstream target protein glycogen synthase kinase-3, thereby inhibiting HIF-1α degradation and promoting its accumulation (Sodhi, Montaner, Miyazaki, & Gutkind, 2001). In addition, AKT has also been reported to promote HIF-1α transcription by activating the mammalian target of rapamycin (mTOR) (Tang & Zhao, 2020). In the present research, H_2_O_2_ treatment significantly elevated the levels of upstream PI3K, total AKT, and p-AKT expression in the early postmortem period (Fig. 4 B, C, D, F, G, and H), which is accompanied by a significant accumulation of HIF-1α (Fig. 4 A and E), indicating that ROS can enhance HIF-1α stabilization through PI3K/AKT signaling pathway.

### 3.6 ROS-accelerated glycolysis contributed to the tenderization of postmortem bovine muscle

**Fig. 5:**
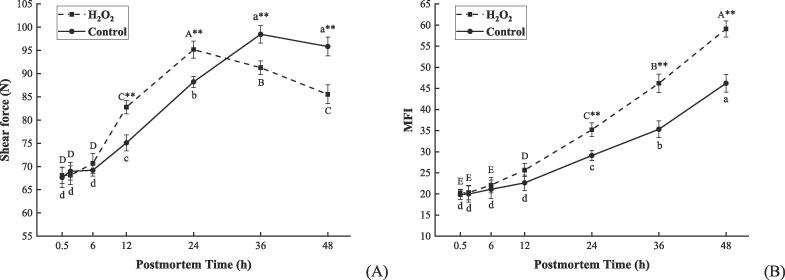
Changes in shear force (A) and MFI (B) of bovine muscle when stored at 4 °C for 0.5, 2, 6, 12, 24, 36, and 48 h after treatment with 200 μM H_2_O_2_ (H_2_O_2_ group) and saline (control group). Data were expressed as mean ± standard error (n = 6). The capital letters and lowercase letters represent the significant difference (*P* < 0.01) in the same group at different times, respectively. ** (*P* < 0.01) represents the difference between the two groups at the same time point, respectively. MFI: myofibril fragmentation index.

As revealed by the presented study, ROS-accelerated glycolysis resulted in a rapid decline in pH (Fig. 2 D). It is noteworthy that a low pH in the early postmortem period promotes the hydrolysis of myofibrillar proteins, resulting in a fast tenderization process during postmortem aging (Baron et al., 2021, Ma et al., 2022). Therefore, shear force and MFI were evaluated in this study to confirm whether ROS affect meat tenderization by accelerating postmortem glycolysis. As shown in Fig. 5 A, both the H_2_O_2_ group and the control group showed a significant increase in shear force at 6–24 h and 6–36 h, respectively (*P* < 0.01), this was regarded as the process of rigor mortis. However, the H_2_O_2_ group reached the maximum shear force (95.18 N) at 24 h, which was 12 h earlier than the control group (98.47 N), indicating that ROS shorten the rigor mortis period. Moreover, shear force decreased significantly at 24–48 h in the H_2_O_2_ group (*P* < 0.01) and was remarkably lower than that in the control group at 36–48 h (*P* < 0.01). MFI is a good indicator of tenderness because it is positively related to the fragmentation degree of myofibrils (Chen et al., 2019). As presented in Fig. 5 B, the H_2_O_2_ group and the control showed a significant increase in the MFI at 6–48 h and 12–48 h, respectively (*P* < 0.01). While the MFI in the H_2_O_2_ group was 1.21 times, 1.31 times, and 1.29 times greater in contrast to the control at 24 h, 36 h, and 48 h, respectively (*P* < 0.01). The above results suggested that ROS promote the tenderization process of the postmortem bovine muscle.

Tenderness is a critical palatability attribute of meat that is improved during postmortem aging (Honikel, 2014). Glycolysis is an important biochemical pathway for the improvement of tenderness, muscle with a fast glycolysis rate shows a lower final shear force (Kamenik et al., 2021, Sun et al., 2019). In this study, ROS-accelerated glycolysis resulted in a rapid decline in pH at 2–12 h in the H_2_O_2_ group (Fig. 2 D), while its shear force increased to the maximum in the following 6–24 h and significantly decreased after 24 h (Fig. 5 A). The above results are supported by Honikel (2014), who indicated that a rapid decline rate in pH shortens the rigor mortis period and subsequently accelerates the tenderization process. Also, our results are consistent with a recent study by Zhao et al. (2022), who indicated that a rapid decline in pH within the first 24 h results in a faster tenderization rate and ultimately lower shear force in bovine muscle. Besides, in the current study, a significantly higher MFI was observed at 24–48 h in the H_2_O_2_ group (Fig. 5 B). This is in line with a recent study by Ma et al. (2022), who found that a higher rate of ROS accumulation, along with the faster decline in pH, is conducive to myofibrillar protein degradation and MFI increase at 12–168 h in postmortem bovine muscle. Based on the finding of Xing et al. (2019), a rapid pH decline leads to an overload of Ca^2+^ in sarcoplasm, which in turn promotes activation of proteases and degradation of myofibrillar proteins. Moreover, a low pH milieu favors to activation of apoptosis-related enzymes, which partly contributes to the improvement of tenderness in postmortem bovine muscle (Honikel, 2014). Together, it is suggested that ROS promote pH decline by accelerating glycolysis, thereby shortening the rigor mortis period and improving meat tenderness.

## 4 Conclusion

This paper investigated the role of ROS in the modulation of glycolysis and tenderization in postmortem bovine muscle. The results indicate that ROS are inevitably generated in the postmortem bovine muscle due to the hypoxic condition, which enhances muscle glycolysis, to a certain extent accompanied by elevated activities of HK and PFK early postmortem. Such a metabolic effect is attributed to the stable HIF-1α accumulation driven by ROS through activating the PI3K/AKT signaling pathway. Notably, treatment with 200 μM exogenous H_2_O_2_ at a ratio of 10:1 (w/v; meat: solution) can lead to a remarkable elevation in the initial levels of ROS and glycolytic potential early postmortem, which is beneficial for shortening the rigor mortis period and subsequently accelerating the tenderization process of bovine muscle. This study provides a new theoretical reference for improving the attributes of meat quality by regulating postmortem glycolysis through the HIF-1α pathway related to ROS.

## CRediT authorship contribution statement

**Cheng Chen:** Conceptualization, Methodology, Writing – original draft. **Zhaobin Guo:** Investigation, Formal analysis, Project administration. **Xixiong Shi:** Validation, Supervision. **Yuxuan Guo:** Visualization. **Guoyuan Ma:** Software. **Jibing Ma:** Writing – review & editing. **Qunli Yu:** Project administration.

## Declaration of Competing Interest

The authors declare that they have no known competing financial interests or personal relationships that could have appeared to influence the work reported in this paper.

## Data Availability

No data was used for the research described in the article.
